# Deliberate dietary adjustments may not mitigate the progression of glaucoma: A two-sample Mendelian randomization study

**DOI:** 10.1097/MD.0000000000042944

**Published:** 2025-07-18

**Authors:** Bin Lin, Long-Long Chen, Meng Xu, Dong-Kan Li

**Affiliations:** a Xiamen Eye Center and Eye Institute of Xiamen University, School of Medicine, Xiamen, China; b Xiamen Clinical Research Center for Eye Diseases, Xiamen, Fujian Province, China; c Xiamen Key Laboratory of Ophthalmology, Xiamen, Fujian Province, China; d Fujian Key Laboratory of Corneal & Ocular Surface Diseases, Xiamen, Fujian Province, China; e Xiamen Key Laboratory of Corneal & Ocular Surface Diseases, Xiamen, Fujian Province, China; f Translational Medicine Institute of Xiamen Eye Center of Xiamen University, Xiamen, Fujian Province, China.

**Keywords:** dietary, protein, carbohydrate, sugar, fat, glaucoma, Mendelian randomization

## Abstract

Ocular diseases, including glaucoma, are significant contributors to visual impairment and irreversible blindness, impacting individuals’ socioeconomic opportunities and quality of life. This study aimed to investigate the causal relationship between dietary components and glaucoma progression. We conducted Mendelian randomization studies to assess the effects of 4 dietary components (protein, carbohydrates, sugar, and fat) on glaucoma. A total of 11,417,548 single nucleotide polymorphisms (SNPs) were screened, and after applying a stringent threshold, we retained relevant SNP markers for each dietary group. We employed 4 regression models: MR-Egger regression, a weighted median estimator, an inverse-variance weighted random-effects model, and a weighted model to analyze the data. The analysis revealed that all obtained *P*-values were greater than .05, indicating no significant association between dietary components and glaucoma. Additionally, sensitivity analyses using the leave-one-out method confirmed the robustness of these findings, showing that the removal of any SNP did not alter the conclusions. The findings suggest that deliberate dietary adjustments may not mitigate the progression of glaucoma. For now, adhering to prescribed medications and considering early surgical intervention when necessary may represent more effective treatments for glaucoma.

## 1. Introduction

Ocular diseases are significant contributors to visual impairment and irreversible blindness, impacting socioeconomic opportunities, quality of life, and even mortality risk.^[1]^ Among these conditions, glaucoma is prominent as a leading cause of irreversible blindness globally, affecting an estimated 76 million individuals.^[2]^ Glaucoma is a group of diseases in which the intraocular pressure rises abnormally, leading to optic nerve damage and irreversible functional decline. Usually, medications or surgical procedures are required to reduce the formation of aqueous humor in the eye or increase its outflow.^[3,4]^ This helps to lower the intraocular pressure and alleviate damage to the optic nerve.

The primary therapeutic focus for glaucoma management revolves around controlling intraocular pressure (IOP).^[5]^ Recently, alternative treatments targeting both IOP-dependent and non-IOP-dependent mechanisms have garnered interest among ophthalmologists^[6]^ and glaucoma patients.^[7]^ A Canadian report suggests that 1 in 9 glaucoma patients explore alternative therapies to manage their condition,^[8]^ including various complementary and alternative medicine practices such as herbal remedies, dietary adjustments, exercise, and body-based therapies.

Research indicates that nutrition may influence IOP or glaucoma through diverse pathophysiological pathways linked to the disease. Nutrition’s potential impact on glaucoma includes antioxidant properties, effects on vascular endothelium, and neuroprotective qualities.^[9]^

Current evidence does not convincingly support the significant beneficial effects of specific dietary interventions on glaucoma outcomes. While dietary modifications are typically considered low-risk and potentially beneficial, these approaches require stronger evidence before being recommended as formal treatments for glaucoma.^[10]^ Despite ongoing research,^[11–14]^ consensus among experts regarding optimal dietary strategies for glaucoma remains elusive.

**Figure 1.**
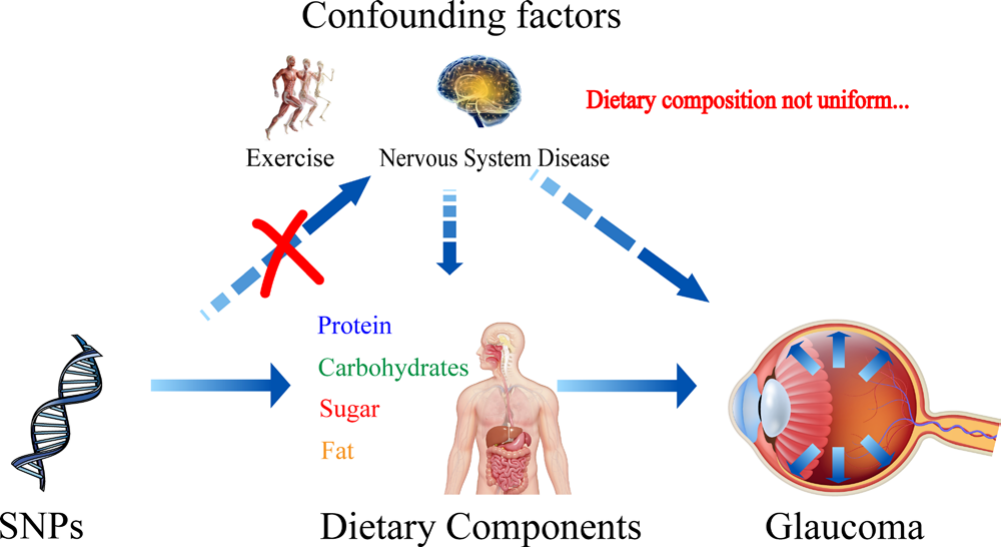
The schematic diagram of this Mendelian randomization study illustrates the process clearly. It demonstrates how Mendelian randomization utilizes representative genome wide association study data from different dietary components and glaucoma to explore their causal relationships. This approach aims to analyze further whether dietary regulation could potentially mitigate the progression of glaucoma.

In recent years, Mendelian randomization studies have gained popularity for their ability to explore precise causal relationships between complex factors.^[15]^ Even the most rigorously designed clinical studies may be subject to confounding factors, especially in research on the relationship between diet and disease. It is extremely challenging to fully control the diets of all subjects to ensure their comparability in a clinical study. In contrast, Mendelian randomization uses single nucleotide polymorphisms (SNPs) as research variables.^[16]^ It attempts to exclude all factors that could potentially influence the study, thus enabling a pure investigation of the causal relationship between dietary components and glaucoma. The principle is shown in Figure 1. In this Mendelian randomization analysis, the null hypothesis is described as stating that the causal effect of changes in dietary components on the onset or progression of glaucoma is zero, that is, there is no causal relationship. If the subsequent analysis results show that all obtained *P*-values are greater than .05, it means that there is not enough evidence to reject the null hypothesis. In other words, it supports the conclusion that there is no significant association between dietary components and glaucoma.

## 2. Methods

We employed Mendelian randomization (MR) to explore how dietary components—protein, sugar, carbohydrates, and fats—potentially causally influence the development of glaucoma. It is noteworthy that the dietary components were categorized in a rather broad manner. Although this approach permits a general exploration of the relationship between diet and glaucoma, it inevitably brings about inherent limitations regarding the specificity of our findings. For instance, a broad categorization might overlook crucial nuances within each dietary component category, potentially masking more precise associations that could exist at a finer level of dietary classification.

MR leverages genetic variants as instrumental variables to estimate causal effects amidst confounding factors. All analyses were conducted in R (version 4.3.3), utilizing specialized packages tailored for MR studies including TwoSampleMR (version 0.5.11) and MR methodology.

### 2.1. Date source

We obtained genome wide association study (GWAS) data for the largest sample size of protein intake, carbohydrate intake, sugar intake, and fat intake (Pubmed ID: 30643258) from the Social Science Genetic Association Consortium (SSGAC) website (https://www.thessgac.org). The GWAS data of Glaucoma (GWAS ID: GCST90011766) from the Integrative Epidemiology Unit OpenGWAS (IEU OpenGWAS) project website (https://gwas.mrcieu.ac.uk). The website was accessed on July 10, 2024. The population sources for both datasets were European, with no gender restrictions. The number of SNPs in the diet dataset was 11,417,548, and the number of SNPs in the glaucoma dataset was 14,219,919.

### 2.2. Instrumental variable criteria

The criteria for SNP selection as instrumental variables were as follows:

1. Instrumental variables were required to exhibit a high correlation with the exposure, indicated by an *F*-statistic exceeding 10, demonstrating substantial correlation.^[17]^
2. SNPs chosen as instrumental variables should not show direct associations with the outcome but should influence it solely through exposure, indicating the absence of genetic pleiotropy. A pleiotropy test was conducted, with a result of *P* ≥ .05 indicating no genetic pleiotropy.
3. Instrumental variables were required to be unrelated to unmeasured confounding factors. SNPs selected for MR adhere to the genetic principle of random allele allocation from parents to offspring, minimizing susceptibility to environmental and postnatal factors.

### 2.3. SNP selection

**Figure 2.**
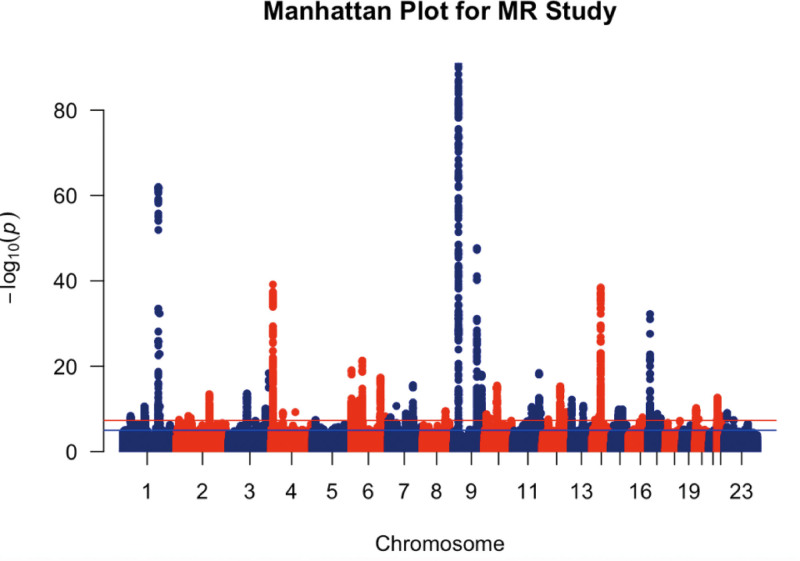
This figure presents a Manhattan plot of glaucoma SNP data. The *x*-axis represents the chromosomal positions across the genome, while the *y*-axis shows the –log 10 (*P*-value), which indicates the significance of the association between each SNP and glaucoma. Notably, the highest values are observed on chromosome 1 and chromosome 9. SNP = single nucleotide polymorphism.

Meaningful SNPs were identified from diet GWAS summary data using stringent selection criteria (*P* < 5 × 10^‐8^). Each SNP’s independence was ensured by applying a linkage disequilibrium coefficient (*r*^2^) threshold of 0.001 and restricting the linkage disequilibrium region width to 10,000 kb, thereby minimizing potential genetic pleiotropy.^[18]^ SNPs associated with dietary factors were extracted from the glaucoma GWAS summary data, with a requirement of *r*^2^ > 0.8 to ensure result accuracy. The identification of potential outliers is achieved by comparing the observed effects with the expected impacts using the Mendelian Randomization Pleiotropy RESidual Sum and Outlier (MR-PRESSO) method. This process is executed through the MR-PRESSO command. Missing SNPs were excluded from the analysis. The Manhattan image of the glaucoma SNP dataset is shown in Figure 2. This image serves as a visual representation of the GWAS results. By observing the plot, it is evident that the values are maximized on chromosome 1 and chromosome 9. These elevated values imply a potentially significant association between the SNPs in these chromosomal regions and glaucoma. Such findings may indicate the presence of genes or genetic regulatory elements on chromosomes 1 and 9 that play crucial roles in the pathogenesis of glaucoma.

### 2.4. Causal relationship validation

To ascertain the causal relationship between diet and glaucoma outcomes using SNPs as instrumental variables, we employed 4 regression models: MR-Egger regression, a weighted median estimator, an inverse-variance weighted random-effects model, and a weighted model. The inverse-variance weighted method computes causal effect estimates directly from summary data, obviating the need for individual-level data. MR-Egger regression constructs a linear model by evaluating the correlation between each SNP and glaucoma (Y) and between each SNP and diet (X). If the intercepts of the MR-Egger regression lines with the *y*-axis are below 0.05, indicating the absence of significant pleiotropic effects in this study.^[19]^ In addition, we will also retest for pleiotropy using MR-PRESSO. The number of repetitions will be set to 10,000. If no outlier data emerges, it suggests that there is no significant pleiotropy in the results of this study.^[20]^ Sensitivity analysis was performed using the leave-one-out approach. All analyses were executed using the TwoSampleMR package (version 0.5.11) within R Studio software (version 4.3.3), with a significance level set at α = .05.

## 3. Results

### 3.1. SNP information screening results

In the dietary SNP dataset, a total of 11,417,548 SNPs were initially obtained. Following filtration based on a threshold of *P* < 5 × 10^‐8^, the protein group retained 265 SNP markers, the carbohydrate group retained 515, the sugar group retained 317, and the fat group retained 264 SNP markers. These SNPs were exported and organized under the TwoSampleMR directory, where sequence names were restructured and arranged accordingly. Subsequently, a clump operation was performed to mitigate genetic pleiotropy effects.

Post-clumping, the protein group retained 7 SNP markers, the carbohydrate group retained 12, the sugar group retained 9, and the fat group retained 5 SNP markers. Following this, the glaucoma dataset was imported and merged with the 4 dietary component datasets, resulting in the final information presented in Table 1. By referring to the Manhattan plot in Figure 2, it is evident that the finally obtained SNP datasets do not overlap with the regions showing the highest values related to the Glaucoma disease. This initially gives the impression that there is no significant causal association between them.

**Table 1:** Summary of the selected SNP information.

Diet	Number	SNP	CHR	BP	A1	Beta	SE
Protein	1	rs13146907	4	39,425,248	A	0.0036	0.0133
	2	rs1461729	8	9,187,242	A	0.0197	0.0209
	3	rs1603978	3	25,108,236	A	0.0319	0.0151
	4	rs445551	2	79,697,982	A	0.0118	0.0139
	5	rs55872725	16	53,809,123	T	0.0263	0.013
	6	rs780094	2	27,741,237	T	‐0.0019	0.0131
	7	rs838133	19	49,259,529	A	0.0061	0.0137
Carbohydrate	1	rs10206338	2	60,209,981	A	0.0046	0.0131
	2	rs10433500	3	85,546,798	A	0.0539	0.0135
	3	rs10510554	3	25,099,776	T	‐0.0169	0.013
	4	rs10962121	9	15,702,704	T	0.012	0.0128
	5	rs1104608	16	73,912,588	C	‐0.0003	0.0134
	6	rs2472297	15	75,027,880	T	0.0523	0.0153
	7	rs36123991	17	44,359,663	T	0.0611	0.0185
	8	rs4420638	19	45,422,946	A	0.0632	0.0177
	9	rs7012637	8	9,173,209	A	0.005	0.0134
	10	rs7190396	16	53,822,502	T	‐0.0213	0.014
	11	rs8097672	18	1,839,601	A	0.0297	0.0181
	12	rs838144	19	49,250,239	T	‐0.0072	0.0131
Sugar	1	rs12713415	2	60,205,134	C	‐0.0069	0.0146
	2	rs12721051	19	45,422,160	C	0.0639	0.0177
	3	rs13202107	6	51,395,463	A	0.0501	0.0157
	4	rs341228	18	6,395,336	T	‐0.0032	0.0137
	5	rs7012814	8	9,173,358	A	0.0049	0.0134
	6	rs7619139	3	25,110,415	A	0.02	0.0131
	7	rs8097672	18	1,839,601	A	0.0297	0.0181
	8	rs838144	19	49,250,239	T	‐0.0072	0.0131
	9	rs9972653	16	53,814,363	T	0.0272	0.013
Fat	1	rs10468280	16	53,827,479	A	‐0.0296	0.013
	2	rs1229984	4	100,239,319	T	0.0299	0.0363
	3	rs33988101	19	49,218,111	T	0.0163	0.0131
	4	rs57193069	7	1,862,417	A	0.0112	0.0135
	5	rs7012814	8	9,173,358	A	0.0049	0.0134

A1 = effector allele, BP = location, CHR = chromosome number, SNP = single nucleotide polymorphism.

### 3.2. Causal relationship verification

**Figure 3.**
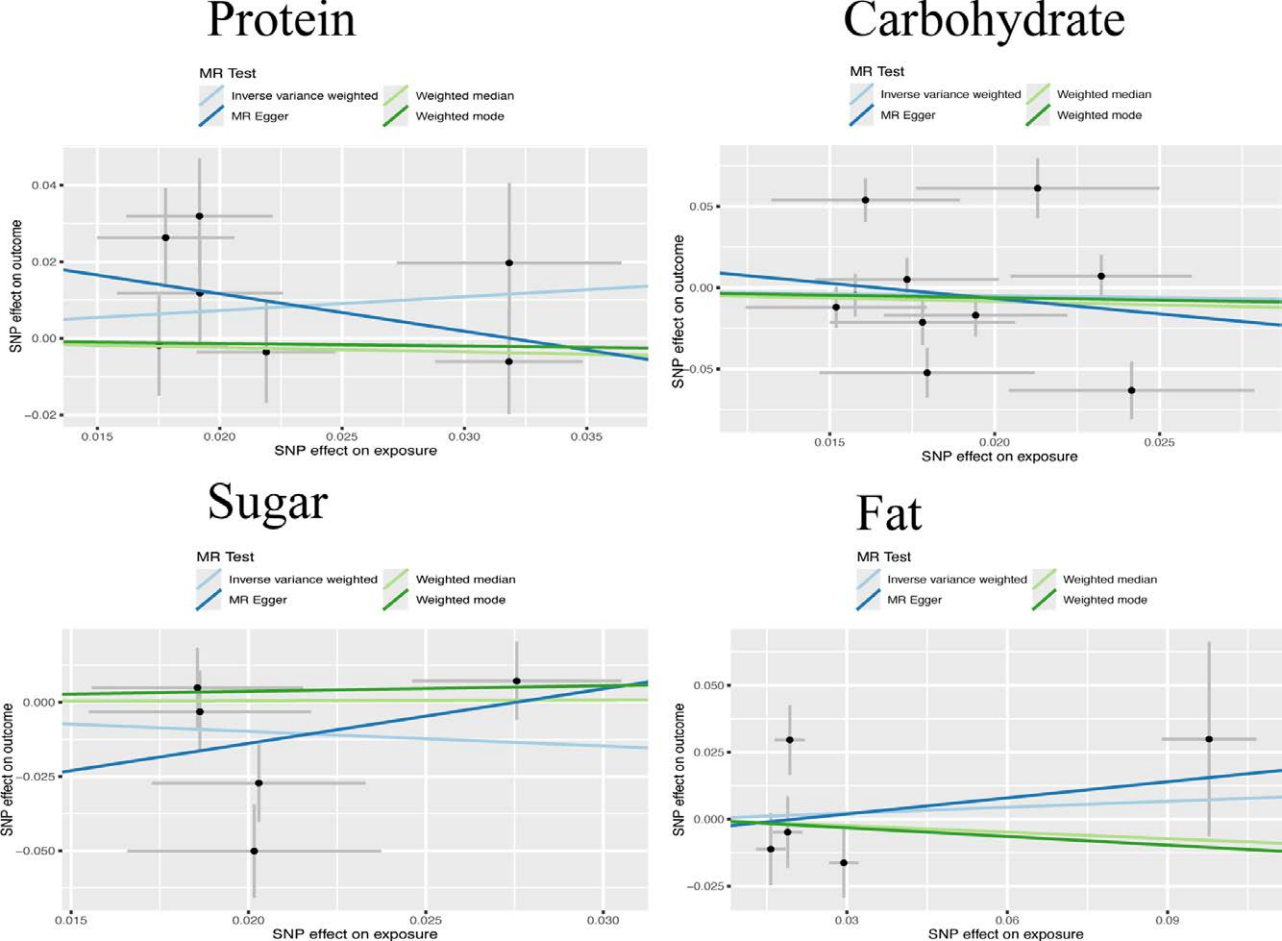
None of the regression equations for the 4 dietary components show a significant association with glaucoma. Additionally, the intercepts of the MR-Egger regression lines with the *y*-axis are all below 0.05, indicating the absence of significant pleiotropic effects in this study.

After conducting Mendelian randomization studies on the effects of 4 dietary components on glaucoma, the regression results depicted in Table 2 indicate that all obtained *P*-values are greater than .05. This suggests that altering dietary components does not significantly influence the treatment or improvement of glaucoma. Even when the number of iterations was set to 10,000 in the MR-PRESSO test, no outlier data was detected. This could potentially be attributed to the relatively small number of SNPs ultimately obtained in each group. Nevertheless, it also provides evidence that there is no significant pleiotropy in the results of this study. The scatter plot illustrating these findings is presented in Figure 3. It can be observed that although some regression formulas exhibit a certain obvious slope when the slope is >1, it indicates a positive influence of the influencing factor, and when the slope is <1, it shows a negative influence. However, by combining with Table 2, we can find that the numerical fluctuations of these slopes are relatively large. That is, the *P*-values are all significantly greater than .05, which fails to prove that these dietary components have any impact on the occurrence and development of glaucoma.

**Table 2:** Regression model results of the 4 methods.

Four methods MR regression model results					
Diet	Method	β	SE	OR (95% CI)	*P*
Protein	MR-Egger	‐0.979	1.093	0.376 (0.044–3.202)	.412
	WME	‐0.118	0.319	0.888 (0.475–1.662)	.711
	IVW	0.363	0.278	1.437 (0.834–2.476)	.191
	Weighted mode	‐0.067	0.377	0.935 (0.447–1.957)	.864
Carbohydrate	MR-Egger	‐1.879	4.06	0.153 (5.347 × 10^‐5^–436.268)	.656
	WME	‐0.422	0.377	0.656 (0.313–1.373)	.263
	IVW	‐0.244	0.594	0.783 (0.244–2.511)	.681
	Weighted mode	‐0.305	0.443	0.737 (0.309–1.757)	.508
Sugar	MR-Egger	1.836	3.286	6.269 (0.010–3926.307)	.615
	WME	0.026	0.387	1.026 (0.480–2.191)	.947
	IVW	‐0.49	0.496	0.613 (0.232–1.621)	.323
	Weighted mode	0.186	0.427	1.204 (0.521–2.782)	.686
Fat	MR-Egger	0.201	0.736	1.222 (0.289–5.169)	.803
	WME	‐0.081	0.306	0.922 (0.506–1.679)	.791
	IVW	0.074	0.338	1.077 (0.556–2.087)	.826
	Weighted mode	‐0.108	0.386	0.898 (0.421–1.913)	.794

IVW = inverse-variance weighted, WME = weighted median estimator.

### 3.3. Sensitivity analysis

**Figure 4.**
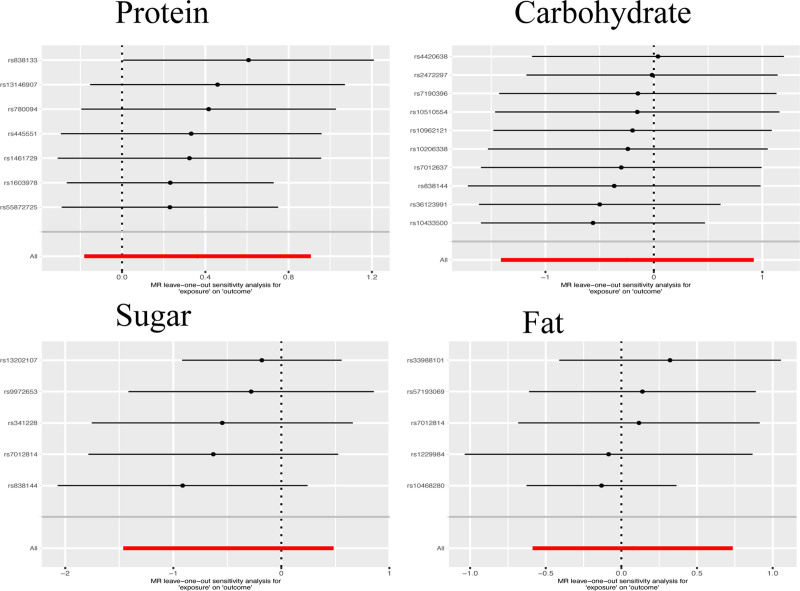
Sensitivity analysis results of the SNP information. All markers observed exhibit a span across the coordinate zero, and the exclusion of any individual marker does not lead to changes in the conclusions. This underscores the robustness of the study’s findings. SNP = single nucleotide polymorphism.

Further sensitivity analysis using the leave-one-out method demonstrated consistent findings: removing any SNP did not alter the conclusions. This robustness indicates the stability of the MR findings in this study. Thus, it further substantiates that patients can not improve their glaucoma condition by modifying the composition of their diet concerning protein, carbohydrates, sugars, and fats. The above is shown in Figure 4.

### 3.4. Heatmap

**Figure 5.**
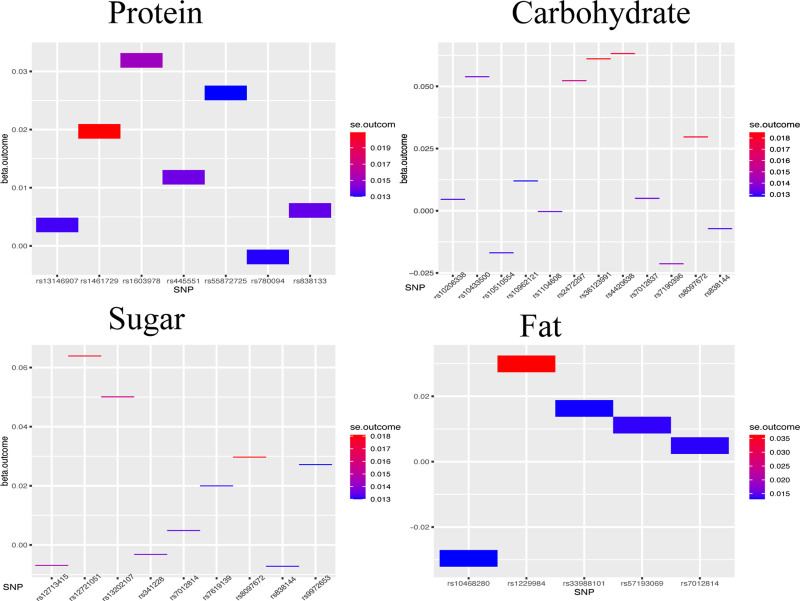
In the heatmap, SNPs are plotted on the *x*-axis, beta values on the *y*-axis, and standard errors are indicated by the color fill. Results shown in blue indicate high reliability and stability, while those in red indicate the opposite. It is observed that in all 4 scenarios, blue lines are distributed around the zero value, indicating that dietary components do not influence the onset or progression of glaucoma. SNP = single nucleotide polymorphism.

We imported the calculated SNP datasets for each group into the R software, generating heatmaps where SNPs were plotted on the *x*-axis, beta values on the *y*-axis, and standard errors used for color filling. In this visual representation, blue bands indicate more accurate and reliable results, while red bands signify lower accuracy. SNP associations close to zero in color intensity suggest minimal impact on the outcome. Which is shown in Figure 5. We can observe that some of the reliable data are very close to the *x*-axis, that is, the value of 0. This indicates that these dietary components have minimal impact on the occurrence and development of glaucoma.

## 4. Discussion

Glaucoma remains a leading cause of irreversible blindness globally, affecting numerous patients who experience visual impairment due to this condition.^[1]^ It is insidious, often progressing asymptomatically and thereby being overlooked until irreversible vision loss occurs, significantly impacting professional activities and daily function.^[21]^

Currently, the primary proven method for effectively managing glaucoma progression is through IOP reduction.^[22]^ Pharmacological therapies primarily target lowering IOP yet are associated with adverse events that may inconvenience patients. Surgical interventions aimed at enhancing aqueous outflow, including trabeculectomy, canaloplasty, or newer minimally invasive techniques,^[23]^ have significantly improved glaucoma control, albeit with potential complications.^[24]^ Consequently, patients often seek alternative treatments,^[25]^ with dietary intervention emerging as a major area of interest. Some studies suggest that dietary composition may influence factors such as intraocular pressure,^[26]^ glaucoma incidence,^[14]^ and disease progression.^[27]^ Encouraging findings regarding certain nutrients have been reported; however, their clinical validation remains challenging due to the need for lifelong administration tailored to patients. Longer follow-up periods and larger sample sizes are required to enhance our understanding of the role of nutrition in glaucoma.^[24]^ Importantly, individuals with a positive family history of glaucoma have a higher risk of developing the condition compared to those without any relatives affected. Interestingly, dietary intake patterns show a heritability rate of approximately 30%.^[28]^

In light of these considerations, our Mendelian randomization study aimed to investigate the causal relationships between dietary protein, carbohydrates, sugars, fats, and glaucoma, while rigorously controlling for confounding factors. Our findings, derived from stringent SNP selection and consolidation, suggest that these dietary components do not significantly influence the onset or progression of glaucoma. Stability analyses and heatmap assessments (Fig. 5) further confirm the reliability and stability of these conclusions.

The verification of the stability and reliability of our research results is multidimensional. By comparing whether the final SNP data overlaps with the high *P*-value regions in the Manhattan plot, jointly validating with multiple regression formulas, and using the heatmap to reflect the influence of dietary factors on the results, it has been confirmed that several dietary components in this study do not have a significant impact on the occurrence and development of glaucoma. Regarding our study’s results, we highlight 2 main points. First, the GWAS data we utilized broadly categorized dietary components as “protein,” “carbohydrate,” “sugar,” and “fat,” without further detailing specific constituents such as omega-3 and omega-6 fatty acids, which are known for their vascular properties and prostaglandin analogs that lower IOP.^[29]^ Sources rich in omega-3 and omega-6 fatty acids include flaxseed oil, fish oil, and walnuts. Low intake of omega-3 fatty acids has been speculated to increase the risk of primary open-angle glaucoma,^[12]^ yet these specific fat components were not represented in our GWAS data. As mentioned in the method section, it should be noted that the dietary components were classified quite broadly in our Mendelian randomization study, which might neglect critical details within each dietary component type. Second, we propose that merely relying on concentrations of these nutrients in food may not be sufficient to alter the outcomes of optic nerve damage in glaucoma patients, potentially failing to achieve effective therapeutic levels. This could contribute to differing conclusions observed in previous clinical studies.^[30]^

Finally, while this study investigated the genetic association between dietary components and glaucoma within a European population database, its findings have important implications for the prevention and treatment of glaucoma related to dietary components in other populations and countries. However, it is important to acknowledge that the lack of analysis of different ethnic groups represents a significant limitation of our research. Moreover, we believe that a future analysis with more granular dietary components is highly necessary. Such an analysis could potentially uncover more precise relationships between specific dietary factors and glaucoma, which might have been overlooked in our current study due to the broad categorization of dietary components. Additionally, relying exclusively on SNP information cannot exclude nongenetically mediated associations between dietary components and glaucoma, such as lifestyle or environmental factors. As Mendelian randomization focuses on genetic pathways, the findings reflect only genetically influenced dietary patterns and do not comprehensively address all potential causal mechanisms. This would contribute to a more in-depth understanding of the role of diet in glaucoma pathogenesis and could offer more targeted dietary recommendations for glaucoma prevention and management.

## 5. Conclusion

Based on the findings of this study, it appears that altering the composition of dietary protein, carbohydrates, sugars, and fats does not currently offer a means to significantly impact the prognosis of glaucoma patients. For now, adhering to prescribed medications and considering early surgical intervention when necessary may represent more effective treatments for glaucoma.

## Acknowledgments

Thanks to Jing Tang for her help in data collection in this study.

## Author contributions

**Data curation:** Bin Lin, Long-Long Chen, Meng Xu.

**Funding acquisition:** Bin Lin.

**Investigation:** Bin Lin.

**Methodology:** Dong-Kan Li.

**Software:** Bin Lin, Long-Long Chen, Meng Xu.

**Supervision:** Dong-Kan Li.

**Writing – original draft:** Bin Lin, Long-Long Chen, Dong-Kan Li.

**Writing – review & editing:** Dong-Kan Li.
